# Supplementary material for: Artificial Intelligence Prediction of Age from Echocardiography as a Marker for Cardiovascular Disease
Source: medRxiv. 2025 Mar 26:2025.03.25.25324627. Preprint. [Version 1] doi: 10.1101/2025.03.25.25324627 (PMC11974980; doi:10.1101/2025.03.25.25324627)
Supplement: 1 [file NIHPP2025.03.25.25324627V1-supplement-1.pdf]

medRxiv preprint doi: https://doi.org/10.1101/2025.03.25.25324627; this version posted March 26, 2025. The copyright holder for this preprint (which was not certified by peer review) is the author/funder, who has granted medRxiv a license to display the preprint in perpetuity.
It is made available under a CC-BY 4.0 International license .

Characteristic	Predicted Age Group			
	<40 years	40–60 years	60–80 years	>80 years
**Male Count (n, %)**	242 (51.49%)	1482 (60.51%)	2899 (61.02%)	897 (48.30%)
**LVEF < 41 (n, %)**	25 (5.32%)	321 (13.11%)	794 (16.71%)	213 (11.47%)
**Aortic Stenosis (n, %)**	0 (0.00%)	23 (0.94%)	335 (7.05%)	434 (23.37%)
**Mitral Regurgitation (n, %)**	59 (12.55%)	630 (25.72%)	1954 (41.13%)	1133 (61.01%)
**Tricuspid Regurgitation (n, %)**	130 (27.66%)	869 (35.48%)	2347 (49.40%)	1282 (69.04%)
**Aortic Regurgitation (n, %)**	20 (4.26%)	204 (8.33%)	961 (20.23%)	712 (38.34%)
**CABG (n, %)**	0 (0.00%)	57 (2.33%)	320 (6.74%)	119 (6.41%)
**Valve Replacement (n, %)**	1 (0.21%)	84 (3.43%)	442 (9.30%)	304 (16.37%)
**Heart Transplant (n, %)**	14 (2.98%)	572 (23.36%)	443 (9.32%)	4 (0.22%)
**Age (years)**	32.79 ± 8.36	52.36 ± 11.04	70.22 ± 10.55	84.98 ± 7.91
**Predicted Age Ensemble**	33.96 ± 3.7	52.45 ± 5.27	70.02 ± 5.64	84.82 ± 3.55
**Predicted Age PLAX**	35.68 ± 5.59	54.47 ± 6.86	69.42 ± 5.12	79.19 ± 3.19
**Predicted Age A2C**	37.42 ± 8.25	54.87 ± 7.91	69.13 ± 6.84	79.16 ± 5.42
**Predicted Age SC**	43.71 ± 7.63	58.49 ± 6.56	67.37 ± 5.27	74.56 ± 4.74
**Predicted Age A4C**	37.4 ± 6.27	56.51 ± 7.19	71.1 ± 5.74	80.89 ± 3.7
**BMI**	25.36 ± 5.53	28.42 ± 8.29	27.19 ± 6.52	25.57 ± 6.95
**Systolic BP (mmHg)**	113.97 ± 20.12	121.16 ± 22.7	122.69 ± 31.23	130.16 ± 47.97
**Diastolic BP (mmHg)**	70.7 ± 13.11	75.13 ± 15.44	69.75 ± 16.28	68.06 ± 15.34
**LVEF (%)**	60.17 ± 10.34	57.32 ± 14.64	55.87 ± 15.74	58.54 ± 13.03
**LVIDd 2D (cm)**	4.78 ± 2.59	4.64 ± 1.35	4.53 ± 0.92	4.28 ± 1
**LVIDs 2D (cm)**	3.26 ± 2.29	3.19 ± 1.17	3.18 ± 1.01	2.91 ± 0.75
**LVPWd 2D (cm)**	0.93 ± 0.38	1.05 ± 0.33	1.1 ± 0.29	1.16 ± 0.41
**LVPWs 2D (cm)**	1.3 ± 0.52	1.39 ± 0.53	1.53 ± 0.36	1.61 ± 0.63
**IVSd 2D (cm)**	0.92 ± 0.4	1.07 ± 0.33	1.14 ± 0.35	1.21 ± 0.29
**IVSs 2D (cm)**	1.42 ± 0.23	1.29 ± 0.34	1.49 ± 0.34	1.58 ± 0.35
**LA Dimension 2D (cm)**	3.24 ± 0.64	3.66 ± 0.81	3.95 ± 0.85	4.12 ± 0.88
**TR Peak Gradient (mmHg)**	19 ± 8.1	23.97 ± 12.08	27.23 ± 11.91	32.17 ± 12.95
**RA Pressure (mmHg)**	3.86 ± 2.41	4.83 ± 3.71	5.69 ± 4.26	5.79 ± 4.18
**PA Pressure (mmHg)**	22.85 ± 8.43	28.6 ± 12.03	33.2 ± 13.71	38.34 ± 14.46
**Total Count (n)**	470	2449	4751	1857

Supplementary Table 1: Imaging characteristics grouped by predicted age group

medRxiv preprint doi: https://doi.org/10.1101/2025.03.25.25324627; this version posted March 26, 2025. The copyright holder for this preprint (which was not certified by peer review) is the author/funder, who has granted medRxiv a license to display the preprint in perpetuity.
It is made available under a CC-BY 4.0 International license .

Disease	ICD-9 Codes	ICD-10 Codes
**CABG**	V45.81, V45.09, 414.01, 414.05, 996.03	Z95.1, Z95.5, I25.10, I25.700, I25.708, I25.709, T82.211A, T82.218A, T82.218D, T82.218S
**Valve Replacement**	V15.1, V42.2, V43.3	Z95.2, Z95.3, Z95.4, Z95.4
**Heart Transplant**	V42.1	Z94.1

Supplementary Table 2: ICD codes used for exclusion criteria.

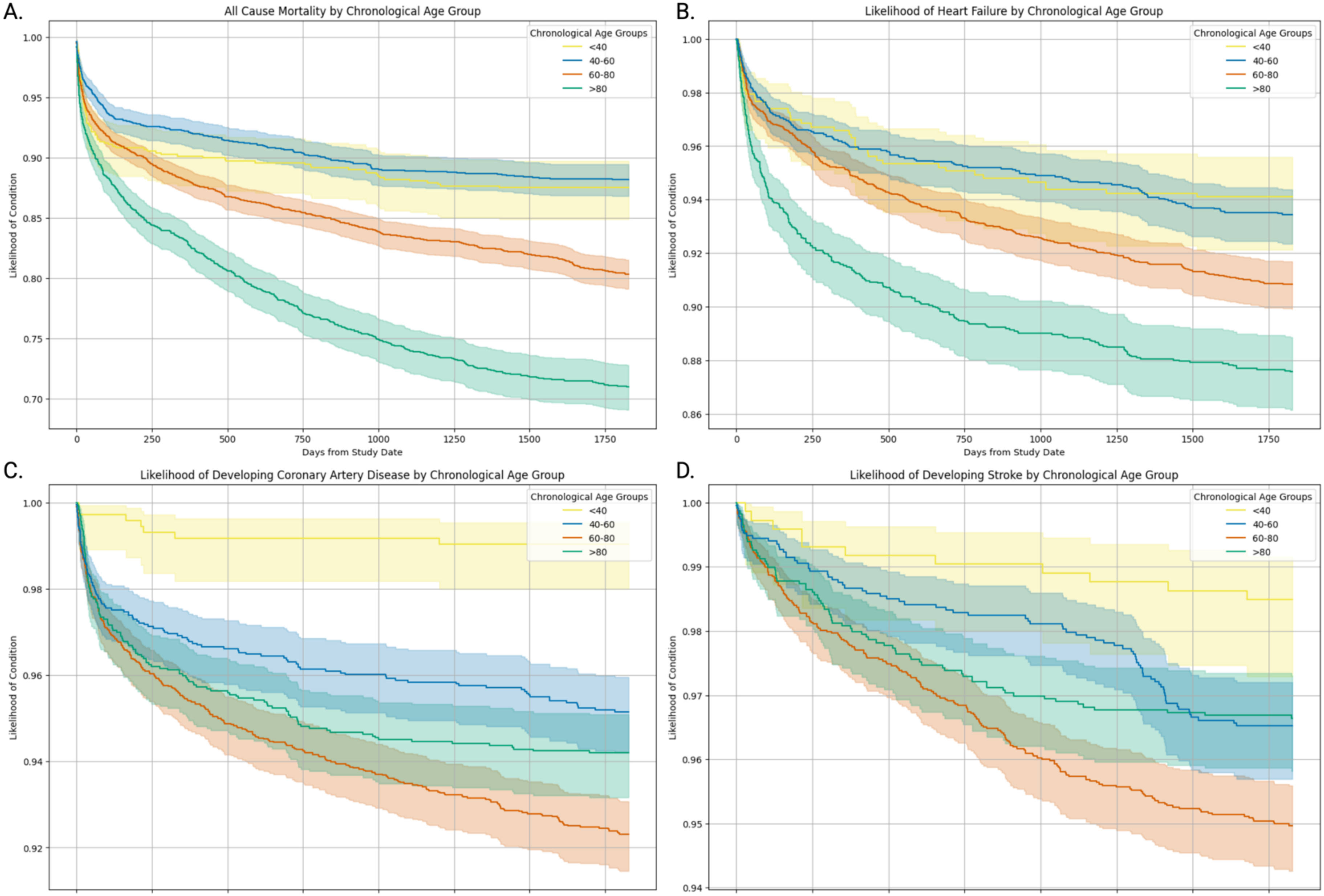

Supplementary Figure 1: Kaplan-Meier survival curves illustrating the association between chronological age group and four clinical endpoints: (A) all-cause mortality, (B) heart failure, (C) coronary artery disease, and (D) stroke

medRxiv preprint doi: https://doi.org/10.1101/2025.03.25.25324627; this version posted March 26, 2025. The copyright holder for this preprint (which was not certified by peer review) is the author/funder, who has granted medRxiv a license to display the preprint in perpetuity.
It is made available under a CC-BY 4.0 International license .

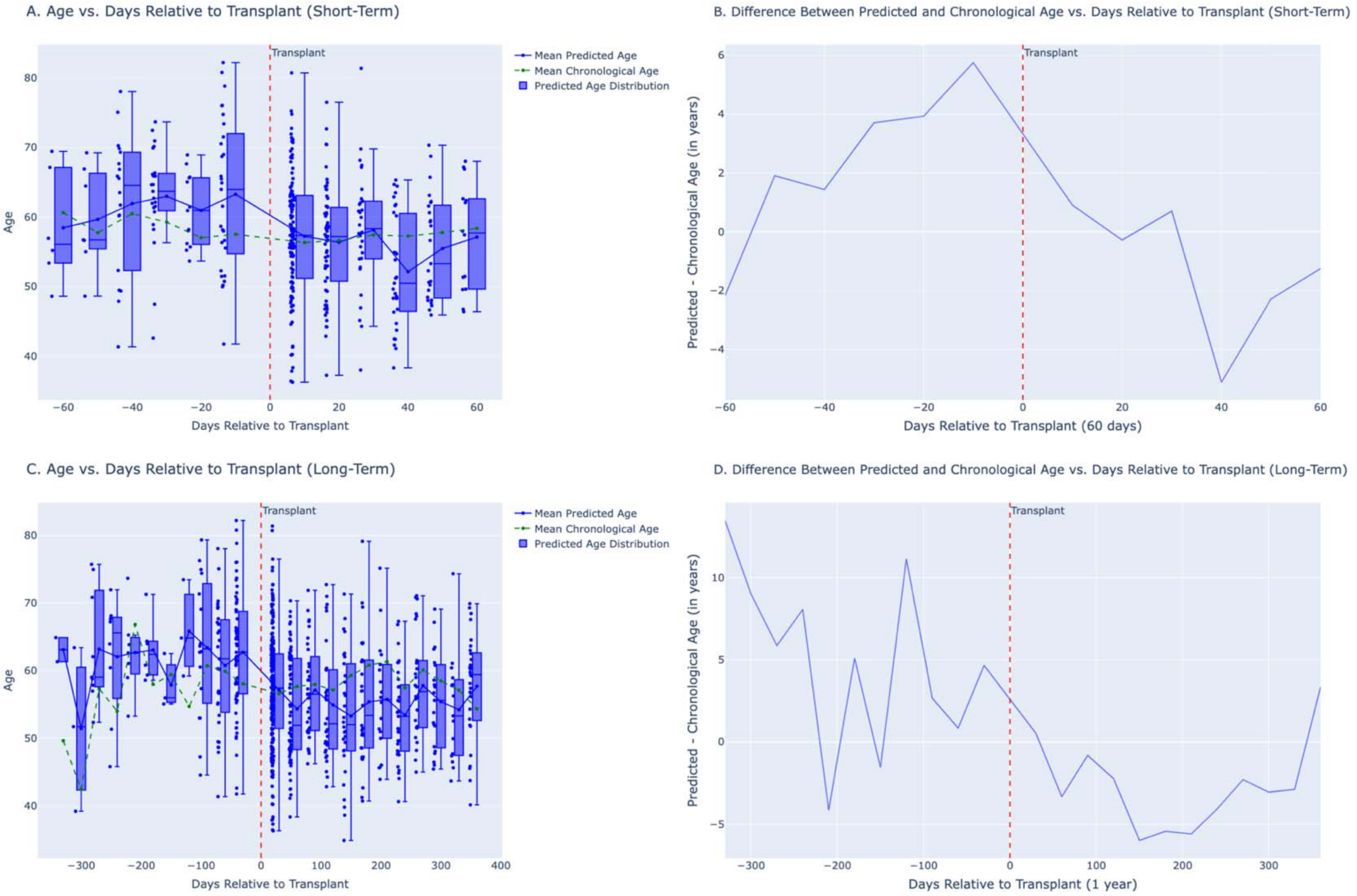

Supplementary Figure 2. Changes in predicted age (left panels) and the difference between predicted and chronological age (right panels) over time relative to the day of transplant (red dashed line at x = 0). Days before transplant are negative on the x-axis, and days after transplant are positive. (A) and (C) show boxplots of predicted age for shorter (A) and extended (C) time windows (60 days, with 10-day interval; one year with 30-day interval). (B) and (D) show how much the predicted age differs from chronological age for shorter (B) and extended (D) time window.

medRxiv preprint doi: https://doi.org/10.1101/2025.03.25.25324627; this version posted March 26, 2025. The copyright holder for this preprint (which was not certified by peer review) is the author/funder, who has granted medRxiv a license to display the preprint in perpetuity.
It is made available under a CC-BY 4.0 International license .
